# *Mycobacterium tuberculosis* carbon and nitrogen metabolic fluxes

**DOI:** 10.1042/BSR20211215

**Published:** 2022-02-04

**Authors:** Ye Xu, Pooja, Khushboo Borah

**Affiliations:** School of Biosciences and Medicine, Faculty of Health and Medical Sciences, University of Surrey, Guildford GU2 7XH, U.K.

**Keywords:** Carbon, Macrophage, Metabolic fluxes, Metabolism, Mycobacterium tuberculosis, Nitrogen

## Abstract

*Mycobacterium tuberculosis* (Mtb) is one of the most formidable pathogens causing tuberculosis (TB), a devastating infectious disease responsible for the highest human mortality and morbidity. The emergence of drug-resistant strains of the pathogen has increased the burden of TB tremendously and new therapeutics to overcome the problem of drug resistance are urgently needed. Metabolism of Mtb and its interactions with the host is important for its survival and virulence; this is an important topic of research where there is growing interest in developing new therapies and drugs that target these interactions and metabolism of the pathogen during infection. Mtb adapts its metabolism in its intracellular niche and acquires multiple nutrient sources from the host cell. Carbon metabolic pathways and fluxes of Mtb has been extensively researched for over a decade and is well-defined. Recently, there has been investigations and efforts to measure metabolism of nitrogen, which is another important nutrient for Mtb during infection. This review discusses our current understanding of the central carbon and nitrogen metabolism, and metabolic fluxes that are important for the survival of the TB pathogen.

## Introduction

Despite decades of research and development in vaccination and therapeutics, tuberculosis (TB) still remains one of the world’s deadliest infectious diseases [1]. TB causes mortality of more than one million people every year. According to the latest World Health Organization global TB report, the number of individuals recovered from TB with treatment and preventative therapies did improve in 2018 and 2019 [1,2] but the COVID-19 pandemic brought major setbacks to the treatment and cure and escalated the burden of this disease [3,4]. Latent TB infection (LTBI), where individuals remain asymptomatic, but with a variable risk of reactivation to active disease, accounts for over a billion cases globally; LTBI remains a problem due to the lack of efficient diagnostic tools and therapies [5,6]. Drug resistance in TB is one of the pressing problems that needs urgent attention. The causative agent of TB, *Mycobacterium tuberculosis* (Mtb) becomes resistant to the first-line drugs isoniazid or rifampicin causing multidrug-resistant (MDR)-TB. Extensively drug-resistant (XDR)-TB cases are the ones where the MDR-TB strains are resistant to any fluoroquinolone and second-line drugs. There were 470000 global incidents, and 180000 deaths from MDR-TB in 2020 [1]. We need to develop new diagnostic tools and treatments to detect, manage and cure TB to fulfil the WHO’s strategy to end TB by 2030. It is important to understand Mtb’s biology during infection to devise effective therapeutics. Metabolism of the TB pathogen is important for its survival and virulence in the human host, and in recent years, Mtb’s metabolism has been intensely researched for anti-TB drug development. There are several excellent studies on different aspects of Mtb’s metabolism in disease, persistence, and in drug development. This review discusses our current understanding of Mtb’s metabolism with prime focus on central carbon and nitrogen metabolism, which are key to sustain metabolic function in any organism.

Metabolism of a biological system is key to sustaining growth, survival, and function. Metabolism comprises complex sets of biological processes with hundreds of biochemical reactions that can be broadly classified into one that produces metabolic products, energy, and biomass (anabolism) and the other involved in breakdown of substrates (catabolism). Cellular carbon metabolism is at the heart of sustaining the metabolic network function. Dysfunction or adaptations in carbon metabolism is implicated in many human diseases including cancer, cardiovascular and metabolic disorders [7]. In cancer, tumour cells adapt to increased glucose uptake and increased glycolytic state known as the ‘Warburg effect’ [7,8] This effect is accompanied with reduced mitochondrial metabolism and oxidative phosphorylation [7,8]. One-carbon metabolism, with glucose converted into serine and subsequently into nucleotides, has been identified as another important hallmark of cancer cells [7,9]. Warburg effect in TB lesions was observed over a decade ago [10] and since then it has been researched as a ‘target’ for developing adjunctive host-directed therapeutics (HDTs) to control Mtb infection [11]. The idea is that upon Mtb infection, infected immune cells adopt an increased glycolytic metabolic state required to mount maximal antibacterial and proinflammatory response [12] and enhancing this Warburg effect could be used to control TB. Several metabolic elements of the Warburg effect that are important for immune cell metabolism or immunometabolism in disease have been investigated and are discussed in detail by other reviews [11,12]. In addition to evoking metabolic changes in the host, the TB pathogen itself undergoes carbon metabolic adaptations to maximize its survival and pathogenicity. The adaptations in Mtb’s carbon metabolism and metabolic fluxes during infection is the focus of this section.

Mtb are transmitted through aerosols and are engulfed by alveolar macrophages in the lungs of infected individuals. Inside macrophages, Mtb pathogen resides in phagosomes where it is challenged with harsh host cell defence responses including hypoxia, acidification, nutrient starvation, and oxidative stress [13–15]. However, Mtb has evolved mechanisms to escape these macrophage antibacterial responses using metabolic adaptations as one of its strategies. Mtb flexibly co-metabolizes multiple carbon substrates inside the host cells and Mtb’s central carbon metabolism (CCM) play key roles in physiology and pathogenicity [16,17]. Several omic-based approaches including genomics, transcriptomics, metabolomics, and fluxomics have revealed the organisation and function of Mtb’s central carbon metabolic network.

## Glycolytic and gluconeogenic carbon metabolism

**Figure 1:**
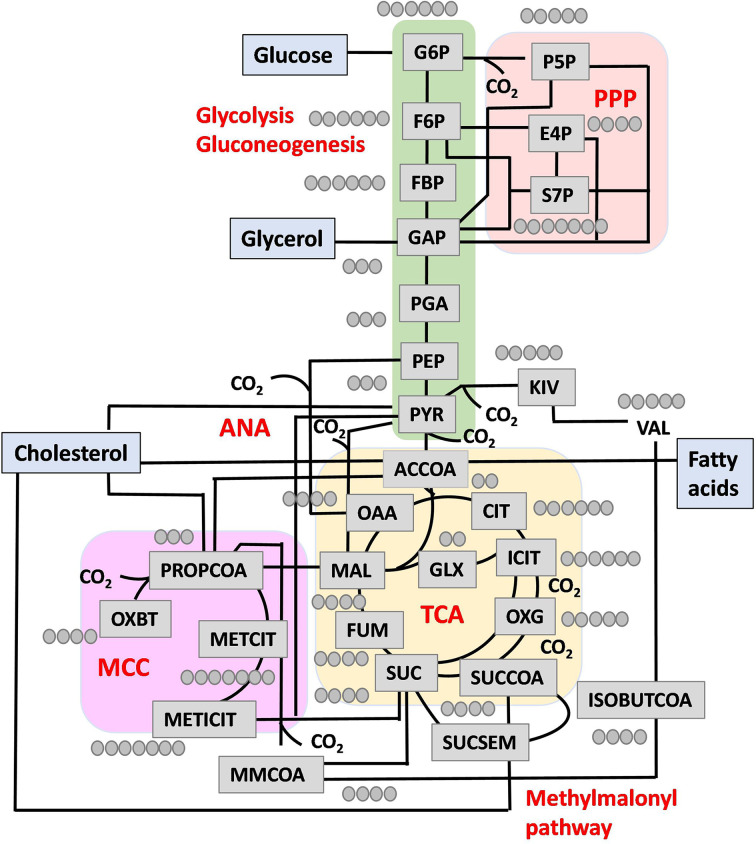
CCM in Mtb The network shows metabolic intermediates; reactions for glycolysis, gluconeogenesis, anaplerosis (ANA), the tricarboxylic acid cycle (TCA), methyl citrate cycle (MCC), methylmalonyl pathway, pentose phosphate pathway (PPP) and glyoxylate shunt; various carbon substrates including glucose, glycerol, cholesterol, and fatty acids; red circles as carbon atom numbers participating in CCM. Metabolites shown are MALOAA (malate + oxaloacetate), SUC (succinate), SUCSEM (succinate semialdehyde), ACCOA, PYR (pyruvate), ICIT (isocitrate), GLX (glyoxylate), OXG (α-ketoglutarate), SUCCOA (succinyl-CoA), FUM (fumarate), G6P (glucose-6-phosphate), F6P (fructose-6-phosphate), FBP (fructose 1,6-bisphosphate), GAP (glyceraldehyde-3-phosphate), PGA (phosphoglyceric acid), PEP (phosphoenolpyruvate), PYR (pyruvate), P5P (pentose-5-phosphate), E4P (erythrose-4-phosphate), and S7P (sedoheptulose-7-phosphate), METCIT (methyl citrate), METICIT (methyl isocitrate), MMCOA (methylmalonyl-CoA), ISOBUTCOA (isobutyl-CoA), OXBT (oxobutanoate). Figure was created with Biorender.com

The genome sequence analysis of Mtb by Cole et al. [18] confirmed the presence of the enzymes of CCM pathways including glycolysis, pentose phosphate pathway (PPP), the tricarboxylic acid cycle (TCA), and glyoxylate shunt (Figure 1). The genes for ATP generation through aerobic oxidative phosphorylation (electron transport chain, cytochrome *b* reductase, cytochrome *c* oxidase) and through anaerobic phosphorylation (nitrate reductase, nitrite reductase, fumarate reductase) are present in Mtb [18,19]. Several studies have demonstrated that Mtb uses a range of glycolytic carbon substrates including sugars and triglycerides *in vitro*, and during early replication in the host [20–25]. Lofthouse et al. [22] conducted a systems-based screen using computational and experimental approaches to compare a range of carbon substrate utilisation in Mtb grown *in vitro* and compared the Mtb profile with its related pathogen *Mycobacterium bovis*, the causative agent of TB in cattle. Mtb utilized carbohydrates including glucose, mannose, trehalose, and two- and three-carbon substrates including glycerol and pyruvate through glycolytic oxidation [22]. In contrast, *M. bovis* was unable to utilise glucose, pyruvate, and alanine due to the mutations in pyruvate kinase *pykA* and alanine dehydrogenase *ald* confirming metabolic heterogeneity between the two mycobacterial pathogens [22]. Mtb has two glucokinases (polyphosphate glucokinase *ppgk* and *glka*) to perform glucose phosphorylation, the first step in glycolysis that incorporates carbon atoms from carbohydrates into the CCM [18,26]. These two glucokinases are important for *in vitro* growth of Mtb on glucose as the carbon source. They are dispensable for Mtb’s intracellular growth but essential for Mtb’s persistence in mice lungs [26]. Mtb’s phosphofructokinase gene *pfkA* catalyses the phosphorylation of fructose 6-phosphate, a key step in glycolysis [18]. Deletion of *pfkA* was non-essential for Mtb’s survival in mice but was essential to sustain the survivability of non-replicating Mtb under hypoxia [27]. Glucose maybe accessible to Mtb in the macrophage intracellular milieu, but it is not the primary carbon source for its intracellular replication [15,23,26,28,29]. *Mycobacterium leprae*, the related pathogen uses host glucose-derived carbon for synthesising amino acids during growth in Schwann cells, but Mtb replicating in human THP-1 macrophages do not [29]. Glycerol is a widely used carbon source for *in vitro* growth of Mtb and precursor for the three-carbon (C3) glycolytic substrates utilized by Mtb inside macrophages [23,25]. Beste et al. [24] provided the first metabolic flux map of Mtb and the vaccine strain *M. bovis* BCG, quantifying the carbon fluxes on glycerol and Tween-80 using Metabolic Flux Analysis (MFA), a systems-based experimental (^13^C-labelling in chemostat system) and computational modelling analyses. Both Mtb and BCG had relatively higher glycolytic/gluconeogenic fluxes at slow and fast growth rates tested. Applying MFA, Beste et al. identified a ‘GAS’ pathway for pyruvate dissimilation involving the oxidative TCA cycle, glyoxylate shunt, and anaplerotic CO_2_ fixation. Isocitrate lyase (*icl*), is an important enzyme for lipid metabolism, for the persistence of Mtb at slow growth rates and for the operation of GAS pathway [24,30–32]. Glycerol metabolism in Mtb modulated the anti-TB drug potency *in vitro* [33,34]. During growth on rich media supplemented with glycerol, the efficacy of Mtb’s cytochrome bc1:aa3 complex inhibitors (imidazopyridine carbox-amide Q203, ND-1088530) was reduced; this was due to the up-regulation of Cyt-bd terminal oxidase as alternate respiratory complex in the presence of the drugs and glycerol, demonstrating that Mtb tunes glycerol utilisation through the CCM and oxidative phosphorylation in order to escape drug killing [34,35].

Pyruvate kinase (*pykA*) is the rate-limiting step of glycolysis and is important for catabolism of glucose, and co-catabolism of carbon sources and fatty acids [20,36]. Deletion of *pykA* did not affect the *in vivo* replication of Mtb in mice models but attenuated *in vitro* utilisation of glycolytic and gluconeogenic substrates through the accumulation of phosphoenolpyruvate (PEP), citrate, aconite, and consequent allosteric inhibition of isocitrate dehydrogenase (*icdh*), a key enzyme of the TCA cycle [18,20,37]. ^13^C-isotopomer analysis and MFA revealed metabolic adaptations of Mtb on bedaquiline (BDQ), an anti-TB drug which inhibits oxidative phosphorylation [36]. MacKenzie et al. demonstrated that the dependence on glycolytic substrate level phosphorylation increases on BDQ and that *pykA* was a key node in this adaptation [36]. BDQ rapidly sterilized a ∆*pykA* Mtb mutant illuminating an effective synergistic drug therapeutic combination of BDQ and inhibitors of *pykA*. Although *pykA* is an attractive target because of its regulatory role on metabolism, the presence of *pykA* human orthologue means that drug development against this Mtb enzyme is not straightforward.

## Anaplerotic node and the TCA cycle fluxes

The anaplerotic or ANA node reactions connect glycolysis, gluconeogenesis, and the TCA cycle (Figure 1). The four enzymes of the Mtb ANA node are phosphoenolpyruvate carboxykinase (PEPCK), pyruvate carboxylase (PCA), malic enzyme (MEZ), and pyruvate phosphate dikinase (PPDK). PEPCK catalyses reversible conversion of oxaloacetate (OAA) into PEP and is essential for the growth of Mtb on fatty acids and for Mtb’s survival in macrophages and mice [38]. Enzymes PCA, PEPCK, and MEZ perform CO_2_ fixation and is important for survival of Mtb in macrophages [23,39]; PEPCK and PPDK are both involved in gluconeogenesis, and PPDK is essential for cholesterol and propionate metabolism [39]. Mtb lacking MEZ displayed altered cell wall composition and attenuated entry into macrophages [39,40]. Mtb lacking PPDK had significantly reduced survival upon BDQ treatment compared with the wildtype posing PPDK as an attractive drug target [36].

The TCA cycle is at the epicentre of CCM that it generates substrates for oxidative phosphorylation and energy production, and biosynthetic precursors for amino acids and lipids. The annotated Mtb’s genome encodes a full TCA cycle [18], but recent years of biochemical analyses has revealed a discontinuous and bifurcated cycle (Figure 1). Tian et al. [41] measured enzymatic activities of citrate synthase, aconitase, isocitrate dehydrogenase, fumarase, malate dehydrogenase and succinate dehydrogenase, key enzymes of the TCA cycle. The activity of α-ketoglutarate dehydrogenase (*kdh*), an enzyme that catalyses conversion of α-ketoglutarate (or 2-oxoglutarate) into succinyl-CoA with production of NADH was lacking in Mtb [41]. Tian et al. [41] posed a variant TCA cycle in Mtb with oxidative and reductive half cycles and identified enzymes including α-ketoglutarate decarboxylase (KDG) (encoded by Rv1248c), GabD1 (encoded by Rv0234c), and GabD2 (encoded by Rv1731) linking the half cycles [41]. KDG catalysed the conversion of α-ketoglutarate into succinate semialdehyde which was then converted into succinate by GabD1/GabD2. Metabolomic analyses showed discontinuous carbon flow through the TCA cycle in between the TCA cycle metabolic intermediates α-ketoglutarate and succinate in Mtb *in vitro* cultures confirming the operation of an alternative route as proposed by Tian et al. [16,25,41]. Glyoxylate shunt is a variant of the TCA cycle and facilitates bypass of carbon oxidation through the oxidative branch of the TCA cycle. Glyoxylate shunt has been demonstrated to be essential for growth of Mtb on fatty acids, acetate, and cholesterol [42–45]. Isocitrate lyase (ICL) and malate synthase (MS), the two enzymes of the glyoxylate shunt facilitates carbon preservation and replenishment of the TCA cycle intermediates through the synthesis of succinate and glyoxylate from isocitrate [18,32,38,46]. Mtb possesses two isoforms of isocitrate lyase genes, *icl1* and *icl2* which are essential for Mtb to grow on fatty acid substrates and to survive in mice models [18,47]. Mtb *icl1* mutant lacked activity of the glyoxylate shunt and methylcitrate cycle and exhibited slow growth on steric acid [37]. In addition to assimilation of fatty acids, glyoxylate shunt also assists Mtb’s survival under hypoxia, oxidative, and antibiotic stress [48–50].

Both the TCA cycle and glyoxylate shunt are primary routes for metabolism of fatty acid-derived substrates. Mtb degrades fatty acids via β-oxidation and generates acetyl-coenzyme A (CoA) which is converted into acetate through the enzymatic activities of phosphotransacetylase (*pta*) and acetate kinase (*ackA*). Acetate can also be converted into acetyl-CoA via acetyl-CoA synthetase (*acs*). Acetate enters the metabolic network via the TCA cycle which is oxidised to generate substrates for ATP production. The use of oxidative or the reductive TCA cycle by Mtb was dependent on the carbon substrate. For example, growth on acetate used the glyoxylate shunt and oxidative TCA cycle, but growth on glycerol used a reductive TCA cycle [43]. Mtb can oxidise lactate to pyruvate using l-lactate dehydrogenase *ildD2*; utilisation of lactate and pyruvate required the TCA cycle, glyoxylate and GABA shunt, valine degradation and methylcitrate cycle [42,51]. During growth on glycerol, Mtb had significantly lower carbon fluxes through the TCA cycle; Mtb used an incomplete TCA cycle along with the alternative GAS pathway involving glyoxylate shunt and anaplerotic CO_2_ fixation [23,45]. In contrast, during growth on cholesterol and acetate, Mtb used a complete TCA cycle with both oxidative and reductive branches, and had significantly higher fluxes through both the TCA cycle and glyoxylate shunt, confirming these two pathways as the primary routes for cholesterol and acetate assimilation [45].

## Methyl citrate cycle fluxes for lipid metabolism

Mtb utilizes host immune cell-derived lipids (fatty acids and cholesterol) as primary nutrient sources for survival in the hypoxic and nutrient-limited macrophage intracellular environment [28,52]. Mtb has a wide array of genes encoding ∼250 enzymes for fatty acid biosynthesis and degradation [18]. Mtb possesses fatty acid synthesis Fas enzyme complexes to synthesize both simple and complex lipids including mycolic acid. Mtb’s Mce1 operon encoding two putative permease subunits (Rv0167/YrbE1A and Rv0168/YrbE1B), six Mce proteins (Rv0169/Mce1A, Rv0170/Mce1B, Rv0171/Mce1C, Rv0172/Mce1D,Rv0173/Mce1E, and Rv0174/Mce1F), and four accessory subunits (Rv0175/Mam1A, Rv0176/Mam1B, Rv0177/Mam1C, and Rv0178/Mam1D) facilitate the transport of fatty acids through the cell envelope [52]. However, the role of Mce1 in the pathogenesis of Mtb remains debatable as there are conflicting studies showing both fitness defects and hypervirulent phenotypes of Mce1 mutants in mice and macrophages, and an anti-inflammatory response inducing phenotype in macrophages [52–54]. Mtb uses *mce4* operon to import host cholesterol, and this operon have been demonstrated to be essential for an optimal growth and persistence of Mtb *in vivo* [28,53]. The *mce4* operon in Mtb comprises two putative, integral membrane permease subunits (Rv3501/YrbE4 and Rv3502/YrbE4B) and six putative cell wall proteins (Rv3499/Mce4A, Rv3498/Mce4B, Rv3497/Mce4C, Rv3496/Mce4D, Rv3495/Mce4E, and Rv3494/Mce4F) [55]. Microarray and gene expression analyses by Santangelo et al. [56] identified the role of Mce3R as a transcriptional regulator controlling the expression of genes for lipid metabolism and β-oxidation in Mtb. Mtb degrades fatty acids using β-oxidation pathways and the precursors derived such as acetyl-CoA is used to fuel central metabolism and lipid biosynthesis. Cholesterol degradation by Mtb yields acetyl-CoA, propionyl-CoA, succinyl-CoA, and pyruvate that enter Mtb’s CCM [57]. Propionyl-CoA derived from cholesterol and fatty acid degradation fuels virulence lipid biosynthesis such as the methyl-branched moieties of phthiocerol-dimycocerosate (PDIM), polyacylated trehalose and sulpholipid (SL) [45,52]. Propionyl-CoA enters CCM through the methyl citrate cycle (MCC) which comprises *prpC*, *prpD*, and *icl* genes (Figure. 1). It is important to maintain the cellular homoeostasis of propionyl-CoA for growth and persistence, as accumulation of this metabolite is toxic to Mtb [58]. In addition to the MCC, methylmalonyl pathway is also operational in Mtb and functions as an alternative pathway for utilisation of propionyl-CoA. Savvi et al. [58] demonstrated that the functionality of the methylmalonyl pathway was dependent on the availability of vitamin B_12_ which served as a cofactor for the enzymatic activity of the *mutAB*-encoded methylmalonyl-CoA mutase. Borah et al. measured the MCC and methylmalonyl pathway fluxes of Mtb growing on cholesterol and acetate (the precursor for fatty acids), and compared these fluxes with that measured during growth on glycerol and oleic acid [45]. Mtb had comparatively reduced MCC fluxes on cholesterol and acetate, as these nutrients were high energy substrates and provided metabolic intermediates that fuelled metabolism and incorporated directly into the biomass. Propionyl-CoA derived from cholesterol degradation was used as the precursor for acylphosphatidylinositol dimannosides (Acyl-PIMs), PIMs, and sulpholipids such as SL-II [45,59–62]. The MCC fluxes were reversed during growth on glycerol, lactate, and pyruvate to synthesize propionyl-CoA as precursor for lipids highlighting the flexible use of the MCC during growth on different carbon substrates [42,45].

## Metabolic fluxes for carbon co-catabolism

*In vitro* growth comparisons on dextrose, acetate, and glycerol and on combinations of substrates (cholesterol-acetate and glycerol-oleic acid) demonstrated Mtb to selectively produce highest biomass on glycerol [25,45]. Such selective use of carbon substrates was also demonstrated in non-pathogenic *Mycobacterium smegmatis*, where carotenoid production was higher on glucose than that on acetate and glycerol [63]. de Carvalho et al. [25] used isotopically labelled ^13^C-substrates to track the incorporation of carbons into the CCM metabolic intermediates of Mtb batch cultures, and demonstrated the use of glycolysis/gluconeogenesis, PPP, and TCA cycle by Mtb during aerobic growth on dextrose, acetate, and glycerol and posed substrate-specific fates and compartmentalised metabolism in Mtb [25]; however, this feature was not observed in a recent work by Borah et al. Mtb cultures grown at metabolic and isotopic steady states in a chemostat system on combinations of ^13^C-labelled substrates (glycerol-Tween 80 or cholesterol-acetate) exhibited no compartmentalised assimilation of different carbon substrates [45]. There were uniform proportions of labelled and unlabelled carbons in the amino acids synthesized from glycerol-Tween 80 and cholesterol-acetate substrates demonstrating no compartmentalised carbon assimilation [45]. The discrepancies between the two studies could be attributed to the metabolic steady state of Mtb, which can be achieved at a controlled growth rate in a chemostat system, but batch culture studies are limited in this respect [25,45]. Mtb showed distinct carbon flux distributions during growth on different carbon substrates and selective use of the CCM fluxes for nutritional flexibility. During growth on glycerol and Tween-80, fluxes through glycolysis and PPP were significantly higher than the TCA cycle and glyoxylate shunt. This profile was reversed during growth on cholesterol and acetate which showed significantly higher TCA cycle and glyoxylate shunt fluxes. Growth on cholesterol and acetate required a conventional MCC for the assimilation of highly reduced carbon units from cholesterol while growth on simple substrates such as glycerol, lactate, and pyruvate required a reverse MCC channelling carbons for the synthesis of propionyl-coenzyme A (CoA) which is a precursor needed for the cell wall synthesis [24,42,45].

## Nitrogen metabolic fluxes

In addition to carbon, nitrogen is another essential building block for biomass including nucleic acids, amino acids, proteins, lipids, and cofactors. Nitrogen metabolism is important for Mtb’s nutrition and survival in the human host. Like other bacterial species, the regulation of nitrogen metabolism in Mtb is dependent on the nitrogen status, i.e., the ratio of the metabolic intermediate α-ketoglutarate or 2-oxoglutarate to glutamine [64]. The regulation occurs at two levels one of which is the transcriptional regulation of genes involved in nitrogen metabolism and the other is post-transcriptional control of the enzymes involved in nitrogen assimilatory pathways [65]. GlnE, GlnB/GlnK, and GlnD are central regulatory proteins for nitrogen metabolism in Mtb [64,66]. GlnR, a transcription regulator protein controls transcriptional and post-transcriptional regulation of genes involved in nitric oxide detoxification and intracellular survival [66,67]. The *amtB-glnK-glnD* operon encoding for AmtB transporter protein, GlnK PII signalling protein and GlnD uridylyl transferase are induced under conditions of nitrogen limitations [68]. GlnE regulates adenylation of glutamine synthetase that catalyses production of glutamine by the ATP-dependent condensation of glutamate and ammonia [66,68]. The glycogen accumulation regulator A (GarA) regulated interconversions between glutamate and 2-oxoglutarate. Phosphorylation of GarA by the serine-threonine protein kinase controls the activity of key nitrogen metabolic enzymes such as glutamate dehydrogenase and glutamine oxoglutarate aminotransferase [68]. Despite the recent progress made in the identification of regulators for nitrogen metabolism, there remain gaps in our complete understanding of the regulatory processes and the steps involved.

*In vitro*, Mtb can utilize a range of nitrogen sources including ammonium chloride and various amino acids [22,69,70]. The genome of Mtb encodes several transporters for nitrogen sources such as AmtB for ammonium chloride, NarK2 for nitrate, and ABC transporters for amino acids [18]. Nitrogen from ammonium is assimilated primarily by the glutamine synthetase/glutamate synthase (GS/GOGAT) pathways [71]. Mtb can also reduce nitrate to ammonium using its nitrate reductase complex comprising *narGHJI* locus [71,72]. Agapova et al. [69] demonstrated that Mtb preferentially utilizes amino acids such as glutamate, aspartate, asparagine, and glutamine over inorganic nitrogen sources *in vitro*. This study also demonstrated that like carbon co-catabolism, Mtb can co-assimilate two amino acids as nitrogen sources *in vitro*. Our own work investigated nitrogen metabolism of Mtb in human macrophages and identified multiple amino acids including aspartate, glutamate, glutamine, valine, leucine, alanine, and glycine that are available to Mtb during intracellular growth [70]. Nitrogen metabolism in Mtb was compartmentalised with some amino acids such as aspartate and glutamine preferentially utilised as nitrogen donors for the synthesis of other amino acids while others such as alanine and glycine were utilised restrictively and incorporated directly into biomass [70]. Aspartate is transported by aspartate transporter AnsP1, which was essential for nitrogen metabolism and survival of Mtb in mice model [73]. Nitrogen from aspartate is assimilated into various amino acids and is used to synthesise biomass. Rv3722, a recently assigned aspartate aminotransferase that facilitated aspartate-dependent nitrogen transfer to form glutamate from 2-oxoglutarate was important for *in vitro* growth and for virulence in mice and macrophages [74]. Asparaginase, *ansA* is essential to assimilate nitrogen from asparagine and to resist acid stress in the phagosomes [75]. Glutamate is *de novo* synthesized primarily via amination of 2-oxoglutarate catalysed by *gltBD* operon, which encodes large and small subunits of GOGAT; glutamate can also be synthesized by glutamate dehydrogenase *gdh*. Deletion of GOGAT and *gdh* causes glutamate auxotrophy in Mtb and significant reduction in growth in presence of glutamate as sole nitrogen source respectively [76]. Glutamine is the primary nitrogen donor for the synthesis of other amino acids in intracellular Mtb [70]. Branched-chain amino acids valine and isoleucine were also used as nitrogen sources by Mtb inside macrophages. Valine was a nitrogen donor for other amino acids; a valine auxotroph was able to survive intracellularly in macrophages demonstrating the direct uptake of valine from the host cells by Mtb [70,77]. Leucine and serine auxotrophs are severely attenuated in macrophages demonstrating the *de novo* biosynthesis of these amino acids is essential in intracellular Mtb, and that the enzymes for their biosynthesis, LeuD and SerC are potential drug targets [70,78]. Alanine and glycine were acquired directly from the host macrophages by Mtb and were incorporated into the biomass such as the cell wall, of which both alanine and glycine are components. Although there has been progress in the identification of amino acids as nitrogen sources for Mtb during infection, the knowledge, identification and functional assignment of transaminases and amino acid transport systems that are important for Mtb’s nitrogen metabolism, survival, and *in vivo* growth remain largely unknown. Mutagenesis and gene knockout analysis studies are useful in identifying those genes that are required for nitrogen uptake or metabolism during intracellular growth, but they cannot provide the nitrogen metabolic flux measurements. To this end, systems-based technology such as MFA and metabolic modelling can aid in quantification of intracellular nitrogen fluxes. However, nitrogen metabolic modelling, isotopic labelling, and flux analysis needs to be further developed. Currently, the incomplete knowledge about the transaminases and lack of nitrogen atomic backbone rearrangement in the metabolic network limits direct application of carbon-based MFA to measure nitrogen fluxes.

## Conclusions and future perspectives

**Figure 2:**
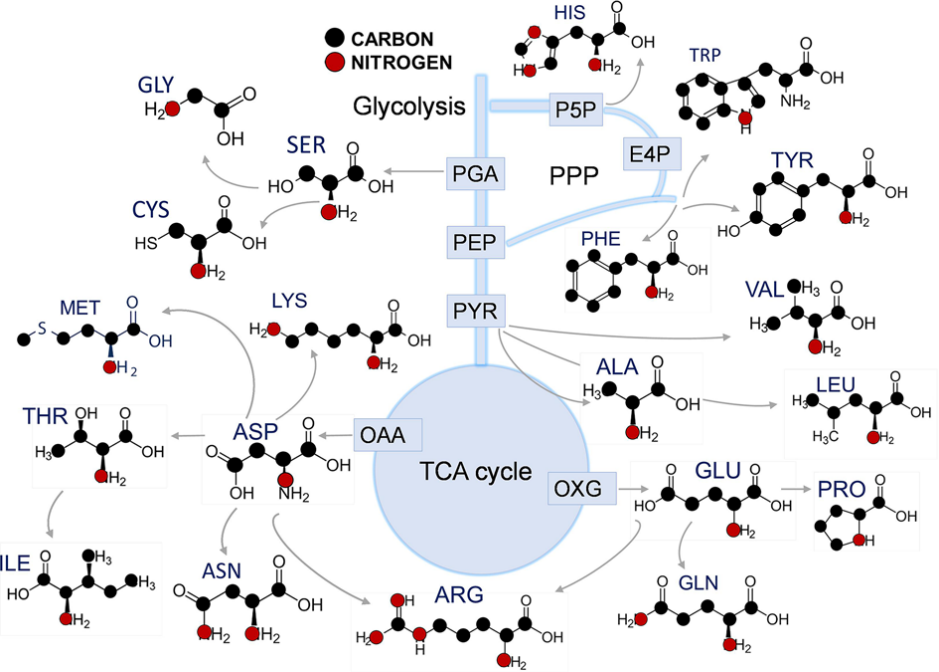
Illustration of carbon–nitrogen co-metabolism for amino acid biosynthesis Amino acids are synthesised from the carbon metabolic intermediates of glycolysis, pentose phosphate pathway (PPP), and the TCA cycle and from nitrogen. Aspartate (ASP) is formed from the amination of oxaloacetate (OAA) produced from the TCA cycle. Glutamate (GLU) is formed from the amination of 2-oxoglutarate or α-ketoglutarate (OXG) produced from the TCA cycle. ASP is the nitrogen and carbon–nitrogen donor to other amino acids including threonine (THR), methionine (MET), lysine (LYS), isoleucine (ILE), and asparagine (ASN). GLU is the carbon–nitrogen donor for proline (PRO) and glutamine (GLN). Both ASP and GLU are precursors for the synthesis of arginine (ARG). Serine (SER) synthesized by the amination of 3-phosphoglyceric acid (PGA); SER is the precursor for glycine (GLY) and cysteine (CYS). Histidine (HIS), phenylalanine (PHE), tyrosine (TYR), and tryptophan (TRP) are synthesised from PPP and glycolytic intermediates (erythrose 4-phosphate (E4P) and phosphoenolpyruvate (PEP)) along with GLU, GLN, or ASP as the nitrogen donor. Valine (VAL), alanine (ALA), and leucine (LEU) are synthesized from the amination of the glycolytic intermediate pyruvate (PYR). Carbon and nitrogen atoms in amino acids are shown as black and red circles respectively. Figure was created with Biorender.com.

Recent decades of research have advanced our understanding of Mtb’s metabolic physiology and identified cellular processes and components that are essential for its virulence and survival in the host. Mtb adapts its nutritional behaviour and metabolic fluxes during infection and growth on different carbon sources. These adaptations have been measured by several studies and attempts to identify metabolic drug targets have been successful. A summary of the enzymes identified as drug targets and their involvement in carbon and nitrogen metabolism is provided in Table 1. Carbon fluxes of Mtb have been extensively researched. Drug-induced metabolic reprogramming and vulnerabilities such as that observed in BDQ-treated Mtb highlighted metabolic targets in the glycolytic substrate-level phosphorylation. Central carbon metabolic enzymes including ICL, PEPCK, PPDK, PYKA are attractive targets for developing anti-TB therapies. Despite the progress in Mtb’s carbon metabolism research, the relevance of the metabolic physiology of Mtb *in vivo* and the validation of the proposed drug targets in clinical trials remain under investigated. Whilst carbon metabolism of Mtb is well-researched, nitrogen metabolism, remains underexplored. Till date, only a few studies exist that identified nitrogen sources such as amino acids to be important for the nutrition and survival of the TB pathogen. The intracellular nitrogen fluxes that support Mtb’s growth *in vitro* or in the human host cells has never been attempted. Also, the intersecting nodes between carbon and nitrogen metabolic pathways, and those that are important for TB infection have not been elucidated. Measuring nitrogen fluxes alone can be technically challenging due to the lack of biochemical information for enzymes such as transaminases/transamidases and the very limited nitrogen atomic backbone rearrangement which is insufficient for robust systems-based analysis such as mathematical modelling and MFA. An alternative approach such as to measure carbon and nitrogen co-metabolic fluxes to overcome the limited atomic measurements for nitrogen and to deduce nitrogen metabolic fluxes from the carbon–nitrogen co-metabolic profiles. An illustration of carbon–nitrogen co-metabolism in amino acids is depicted in Figure 2. Such an approach will identify metabolic nodes and enzymes which are important for sustaining both carbon–nitrogen metabolism. Drugs targeting these nodes or enzymes may be more potent than targeting carbon or nitrogen metabolism alone. It is also important to carefully consider the metabolic drug targets as the drug development may be challenging due to the presence of human orthologs. The relevance of the metabolic physiology measured using drug susceptible Mtb strains needs to be cross-checked with the drug-resistant strains. This is important to extend the identification of drug targets to MDR- and XDR-TB. Most of the metabolic focus research in Mtb was conducted in *in vitro* and in *ex vivo* Mtb replicating in macrophages. Also, the metabolic flux measurement techniques used by previous studies are not consistent across *in vitro* and *ex vivo* models, which makes it difficult to compare the phenotypes derived from the two models. The metabolic flux studies in *in vitro* Mtb primarily uses steady state cultivation and isotopic labelling of the bacteria such as in chemostat setup [45]. However, this is very challenging in case of *ex vivo* Mtb because growth of Mtb-infected human macrophages or cells cannot be cultivated in the *in vitro* chemostat setup. This will require a sophisticated bioreactor setup for cultivation of human cells to provide an appropriate environment for human cell proliferation. Future research to measure metabolic fluxes of Mtb in animal models, and in human tissues such as the lungs will provide new information on the clinically relevant metabolism of Mtb, which in turn will facilitate the development of new and effective therapeutics.

**Table 1:** Summary of Mtb metabolic enzymes that have been used as drug targets or have been identified as potential drug targets

Enzyme targets	Genes	Participation in metabolism
ATP synthase (AtpE)	*Rv1305*	Oxidative phosphorylation (OXPHOS) and energy metabolism (carbon metabolism) [79]
Pyruvate kinase (PykA)	*Rv1617*	Glycolysis (carbon metabolism) [36]
Phosphoenolpyruvate carboxykinase (PEPCK)	*Rv0211*	Gluconeogenesis (carbon metabolism) [39]
Pyruvate phosphate dikinase (PPDK)	*Rv1127c*	Glycolysis/gluconeogenesis (carbon metabolism) [39]
Isocitrate lyase (ICL1)	*Rv0467*	Glyoxylate shunt; methyl citrate cycle (carbon metabolism) [44]
Mce4 operon	*Rv3499c, Rv3494c, Rv3496c, Rv3497c, Rv3498c, Rv3495c, Rv3498c*	Lipid metabolism (carbon metabolism) [28,53]
Asparaginase (AnsA)	*Rv1538c*	Asparagine catabolism (nitrogen metabolism) [75]
Aspartate aminotransferase	*Rv3722*	Aspartate biosynthesis (nitrogen metabolism) [74]
3-isopropylmalate dehydratase (small subunit) (LeuD)	*Rv2987c*	Leucine biosynthesis (nitrogen metabolism) [78]
Phosphoserine aminotransferase (SerC)	*Rv0884c*	Serine biosynthesis (nitrogen metabolism) [70]

The table shows the participation of each enzyme and its respective genes in carbon and nitrogen metabolism. Deletion of these enzymes results in intracellular and *in vivo* growth and survival defects.

## Open Access

Open access for this article was enabled by the participation of University of Surrey in an all-inclusive *Read & Publish* pilot with Portland Press and the Biochemical Society under a transformative agreement with JISC.

## Abbreviations

BDQ: bedaquiline
CCM: central carbon metabolism
GarA: glycogen accumulation regulator A
ICL: isocitrate lyase
KDG: α-ketoglutarate decarboxylase
LTBI: latent TB infection
MCC: methyl citrate cycle
MDR: multidrug-resistant
MEZ: malic enzyme
MFA: Metabolic Flux Analysis
Mtb: *Mycobacterium tuberculosis*
PCA: pyruvate carboxylase
PEPCK: phosphoenolpyruvate carboxykinase
PPDK: pyruvate phosphate dikinase
PPP: pentose phosphate pathway
TB: tuberculosis
TCA: tricarboxylic acid cycle
XDR: extensively drug-resistant

## References

[1] World Health Organization (2020) Global Tuberculosis Report 2020, World Health Organization, Geneva, Licence: CC BY-NC-SA 3.0 IGO. ISBN 978-92-4-001313-1

[2] Tharakan S.M. (2018) Global trends: Tuberculosis. Congressional Research service. 7-5700, https://sgp.fas.org/crs/row/IF11057.pdf

[3] Khurana A.K. and Aggarwal D. (2020) The (in)significance of TB and COVID-19 co-infection. Eur. Respir. J. 56, 2002105 10.1183/13993003.02105-2020 32554537 PMC7301834

[4] Hogan A.B., Jewell B.L., Sherrard-Smith E., Vesga J.F., Watson O.J., Whittaker C. et al. (2020) Potential impact of the COVID-19 pandemic on HIV, tuberculosis, and malaria in low-income and middle-income countries: a modelling study. Lancet Glob. Health 8, e1132–e1141 10.1016/S2214-109X(20)30288-6 32673577 PMC7357988

[5] Blumberg H.M. and Ernst J.D. (2016) The challenge of latent TB infection. JAMA 316, 931–933 10.1001/jama.2016.11021 27599327 PMC5319563

[6] Cohen A., Mathiasen V.D., Schön T. and Wejse C. (2019) The global prevalence of latent tuberculosis: a systematic review and meta-analysis. Eur. Respir. J. 54, 1900655 10.1183/13993003.00655-2019 31221810

[7] Ducker G.S. and Rabinowitz J.D. (2017) One-carbon metabolism in health and disease. Cell Metab. 25, 27–42 10.1016/j.cmet.2016.08.009 27641100 PMC5353360

[8] Rosenzweig A., Blenis J. and Gomes A.P. (2018) Beyond the warburg effect: how do cancer cells regulate one-carbon metabolism? Front. Cell Dev. Biol. 6, 90 10.3389/fcell.2018.00090 30159313 PMC6103474

[9] Newman A.C. and Maddocks O.D.K. (2017) One-carbon metabolism in cancer. Br. J. Cancer 116, 1499–1504 10.1038/bjc.2017.118 28472819 PMC5518849

[10] Kim I.J., Lee J.S., Kim S.J., Kim Y.K., Jeong Y.J., Jun S. et al. (2008) Double-phase 18F-FDG PET-CT for determination of pulmonary tuberculoma activity. Eur. J. Nucl. Med. Mol. Imaging 35, 808–814 10.1007/s00259-007-0585-0 18097664

[11] Cumming B.M., Pacl H.T. and Steyn A.J.C. (2015) Metabolic plasticity of central carbon metabolism protects mycobacteria. Proc. Natl. Acad. Sci. U.S.A. 112, 13135–13136 10.1073/pnas.1518171112 26483480 PMC4629367

[12] Shi L., Eugenin E.A. and Subbian S. (2016) Immunometabolism in tuberculosis. Front. Immunol. 7, 150 10.3389/fimmu.2016.00150 27148269 PMC4838633

[13] Zhai W., Wu F., Zhang Y., Fu Y. and Liu Z. (2019) The immune escape mechanisms of *Mycobacterium tuberculosis*. Int. J. Mol. Sci. 20, 34 10.3390/ijms20020340 PMC6359177 30650615

[14] Queval C.J., Brosch R. and Simeone R. (2017) The macrophage: a disputed fortress in the battle against *Mycobacterium tuberculosis*. Front. Microbiol. 8, 2284 10.3389/fmicb.2017.02284 29218036 PMC5703847

[15] Warner D.F. (2015) *Mycobacterium tuberculosis* metabolism. Cold Spring Harb. Perspect. Med. 5, a021121 10.1101/cshperspect.a021121 PMC4382733 25502746

[16] Rhee K.Y., de Carvalho L.P.S., Bryk R., Ehrt S., Marrero J., Park S.W. et al. (2011) Central carbon metabolism in *Mycobacterium tuberculosis:* an unexpected frontier. Trends Microbiol. 19, 307–314 10.1016/j.tim.2011.03.008 21561773 PMC3601588

[17] Baughn A.D. and Rhee K.Y. (2014) Metabolomics of central carbon metabolism in *Mycobacterium tuberculosis*. Microbiol. Spectrum 2, 1–16 10.1128/microbiolspec.MGM2-0026-2013 26103978

[18] Cole S.T., Brosch R., Parkhill J., Garnier T., Churcher C., Harris D. et al. (1998) Deciphering the biology of *Mycobacterium tuberculosis* from the complete genome sequence. Nature 393, 537–544 10.1038/31159 9634230

[19] Sohaskey C.D. and Wayne L.G. (2003) Role of narK2X and narGHJI in hypoxic upregulation of nitrate reduction by *Mycobacterium tuberculosis*. J. Bacteriol. 185, 7247–7256 10.1128/JB.185.24.7247-7256.2003 14645286 PMC296237

[20] Noy T., Vergnolle O., Hartman T.E., Rhee K.Y., Jacobs W.R., Berney M. et al. (2016) Central role of pyruvate kinase in carbon co-catabolism of *Mycobacterium tuberculosis*. J. Biol. Chem. 291, 7060–7069 10.1074/jbc.M115.707430 26858255 PMC4807288

[21] Beste D.J.V., Espasa M., Bonde B., Kierzek A.M., Stewart G.R. and McFadden J. (2009) The genetic requirements for fast and slow growth in mycobacteria. PLoS ONE 4, e5349 10.1371/journal.pone.0005349 19479006 PMC2685279

[22] Lofthouse E.K., Wheeler P.R., Beste D.J.V., Khatri B.L., Wu H., Mendum T.A. et al. (2013) Systems-based approaches to probing metabolic variation within the *Mycobacterium tuberculosis* complex. PLoS ONE 8, e75913 10.1371/journal.pone.0075913 24098743 PMC3783153

[23] Beste D.J.V., Nöh K., Niedenführ S., Mendum T.A., Hawkins N.D., Ward J.L. et al. (2013) ^13^C-flux spectral analysis of host-pathogen metabolism reveals a mixed diet for intracellular *Mycobacterium tuberculosis*. Chem. Biol. 20, 1012–1021 10.1016/j.chembiol.2013.06.012 23911587 PMC3752972

[24] Beste D.J.V., Bonde B., Hawkins N., Ward J.L., Beale M.H., Noack S. et al. (2011) ^13^C metabolic flux analysis identifies an unusual route for pyruvate dissimilation in mycobacteria which requires isocitrate lyase and carbon dioxide fixation. PLoS Pathog. 7, e1002091 10.1371/journal.ppat.1002091 21814509 PMC3141028

[25] de Carvalho L.P.S., Fischer S.M., Marrero J., Nathan C., Ehrt S. and Rhee K.Y. (2010) Metabolomics of *Mycobacterium tuberculosis* reveals compartmentalized co-catabolism of carbon substrates. Chem. Biol. 17, 1122–1131 10.1016/j.chembiol.2010.08.009 21035735

[26] Marrero J., Trujillo C., Rhee K.Y. and Ehrt S. (2013) Glucose phosphorylation is required for *Mycobacterium tuberculosis* persistence in mice. PLoS Pathog. 9, e1003116 10.1371/journal.ppat.1003116 23326232 PMC3542180

[27] Phong W.Y., Lin W., Rao S.P.S., Dick T., Alonso S. and Pethe K. (2013) Characterization of phosphofructokinase activity in *Mycobacterium tuberculosis* reveals that a functional glycolytic carbon flow is necessary to limit the accumulation of toxic metabolic intermediates under hypoxia. PLoS ONE 8, e56037 10.1371/journal.pone.0056037 23409118 PMC3567006

[28] Pandey A.K. and Sassetti C.M. (2008) Mycobacterial persistence requires the utilization of host cholesterol. Proc. Natl. Acad. Sci. U.S.A. 105, 4376–4380 10.1073/pnas.0711159105 18334639 PMC2393810

[29] Borah K., Girardi K.D.C.V., Mendum T.A., Lery L.M.S., Beste D.J.V., Lara F.A. et al. (2019) Intracellular *Mycobacterium leprae* utilizes host glucose as a carbon source in schwann cells. mBio 10, e02351–e02419 10.1128/mBio.02351-19 31848273 PMC6918074

[30] Pham T.V., Murkin A.S., Moynihan M.M., Harris L., Tyler P.C., Shetty N. et al. (2017) Mechanism-based inactivator of isocitrate lyases 1 and 2 from *Mycobacterium tuberculosis*. Proc. Natl. Acad. Sci. U.S.A. 114, 7617–7622 10.1073/pnas.1706134114 28679637 PMC5530696

[31] Bhusal R.P., Jiao W., Kwai B.X.C., Reynisson J., Collins A.J., Sperry J. et al. (2019) Acetyl-CoA-mediated activation of *Mycobacterium tuberculosis* isocitrate lyase 2. Nat. Commun. 10, 4639 10.1038/s41467-019-12614-7 31604954 PMC6788997

[32] Puckett S., Trujillo C., Wang Z., Eoh H., Ioerger T.R., Krieger I. et al. (2017) Glyoxylate detoxification is an essential function of malate synthase required for carbon assimilation in *Mycobacterium tuberculosis*. Proc. Natl. Acad. Sci. U.S.A. 114, E2225–E2232 10.1073/pnas.1617655114 28265055 PMC5358392

[33] Pethe K., Sequeira P.C., Agarwalla S., Rhee K., Kuhen K., Phong W.Y. et al. (2010) A chemical genetic screen in *Mycobacterium tuberculosis* identifies carbon-source-dependent growth inhibitors devoid of *in vivo* efficacy. Nat. Commun. 1, 57 10.1038/ncomms1060 20975714 PMC3220188

[34] Kalia N.P., Shi Lee B., Ab Rahman N.B., Moraski G.C., Miller M.J. and Pethe K. (2019) Carbon metabolism modulates the efficacy of drugs targeting the cytochrome bc 1 :aa 3 in *Mycobacterium tuberculosis*. Sci. Rep. 9, 8608 10.1038/s41598-019-44887-9 31197236 PMC6565617

[35] Lee B.S., Kalia N.P., Jin X.E.F., Hasenoehrl E.J., Berney M. and Pethe K. (2019) Inhibitors of energy metabolism interfere with antibiotic-induced death in mycobacteria. J. Biol. Chem. 294, 1936–1943 10.1074/jbc.RA118.005732 30530783 PMC6369303

[36] Mackenzie J.S., Lamprecht D.A., Asmal R., Adamson J.H., Borah K., Beste D.J.V. et al. (2020) Bedaquiline reprograms central metabolism to reveal glycolytic vulnerability in *Mycobacterium tuberculosis*. Nat. Commun. 11, 6092 10.1038/s41467-020-19959-4 33257709 PMC7705017

[37] Lee W., VanderVen B.C., Walker S. and Russell D.G. (2017) Novel protein acetyltransferase, Rv2170, modulates carbon and energy metabolism in *Mycobacterium tuberculosis*. Sci. Rep. 7, 72 10.1038/s41598-017-00067-1 28250431 PMC5428333

[38] Marrero J., Rhee K.Y., Schnappinger D., Pethe K. and Ehrt S. (2010) Gluconeogenic carbon flow of tricarboxylic acid cycle intermediates is critical for *Mycobacterium tuberculosis* to establish and maintain infection. Proc. Natl. Acad. Sci. U.S.A. 107, 9819–9824 10.1073/pnas.1000715107 20439709 PMC2906907

[39] Basu P., Sandhu N., Bhatt A., Singh A., Balhana R., Gobe I. et al. (2018) The anaplerotic node is essential for the intracellular survival of *Mycobacterium tuberculosis*. J. Biol. Chem. 293, 5695–5704 10.1074/jbc.RA118.001839 29475946 PMC5900758

[40] Burley K.H., Cuthbert B.J., Basu P., Newcombe J., Irimpan E.M., Quechol R. et al. (2021) Structural and molecular dynamics of *Mycobacterium tuberculosis* malic enzyme, a potential anti-TB drug target. ACS Infect. Dis. 7, 174–188 10.1021/acsinfecdis.0c00735 33356117 PMC8083904

[41] Tian J., Bryk R., Itoh M., Suematsu M. and Nathan C. (2005) Variant tricarboxylic acid cycle in *Mycobacterium tuberculosis:* identification of α-ketoglutarate decarboxylase. Proc. Natl. Acad. Sci. U.S.A. 102, 10670–10675 10.1073/pnas.0501605102 16027371 PMC1180764

[42] Serafini A., Tan L., Horswell S., Howell S., Greenwood D.J., Hunt D.M. et al. (2019) *Mycobacterium tuberculosis* requires glyoxylate shunt and reverse methylcitrate cycle for lactate and pyruvate metabolism. Mol. Microbiol. 112, 1284–1307 10.1111/mmi.14362 31389636 PMC6851703

[43] Murima P., Zimmermann M., Chopra T., Pojer F., Fonti G., Peraro M.D. et al. (2016) A rheostat mechanism governs the bifurcation of carbon flux in mycobacteria. Nat. Commun. 7, 12527 10.1038/ncomms12527 27555519 PMC4999502

[44] Mckinney J.D., Höner zu Bentrup K., Muñoz-Elías E.J., Miczak A., Chen B. et al. (2000) Persistence of *Mycobacterium tuberculosis* in macrophages and mice requires the glyoxylate shunt enzyme isocitrate lyase. Nature 406, 735–738 10.1038/35021074 10963599

[45] Borah K., Mendum T.A., Hawkins N.D., Ward J.L., Beale M.H., Larrouy‐Maumus G. et al. (2021) Metabolic fluxes for nutritional flexibility of *Mycobacterium tuberculosis*. Mol. Syst. Biol. 17, e10280 10.15252/msb.202110280 33943004 PMC8094261

[46] Muñoz-elías E.J., Upton A.M. and Mckinney J.D. (2006) Role of the methylcitrate cycle in *Mycobacterium tuberculosis* metabolism, intracellular growth, and virulence. Mol. Microbiol. 60, 1109–1122 10.1111/j.1365-2958.2006.05155.x 16689789

[47] Muñoz-Elías E.J. and Mckinney J.D. (2005) *M. tuberculosis* isocitrate lyases 1 and 2 are jointly required for *in vivo growth* and virulence. Nat. Med. 11, 638–644 10.1038/nm1252 15895072 PMC1464426

[48] Ahn S., Jung J., Jang I.A., Madsen E.L. and Park W. (2016) Role of glyoxylate shunt in oxidative stress response. J. Biol. Chem. 291, 11928–11938 10.1074/jbc.M115.708149 27036942 PMC4882458

[49] Eoh H. and Rhee K.Y. (2014) Methylcitrate cycle defines the bactericidal essentiality of isocitrate lyase for survival of *Mycobacterium tuberculosis* on fatty acids. Proc. Natl. Acad. Sci. U.S.A. 111, 4976–4981 10.1073/pnas.1400390111 24639517 PMC3977286

[50] Eoh H. and Rhee K.Y. (2013) Multifunctional essentiality of succinate metabolism in adaptation to hypoxia in *Mycobacterium tuberculosis*. Proc. Natl. Acad. Sci. U.S.A. 110, 554–6559 10.1073/pnas.1219375110 PMC3631649 23576728

[51] Billig S., Schneefeld M., Huber C., Grassl G.A., Eisenreich W. and Bange F.C. (2017) Lactate oxidation facilitates growth of *Mycobacterium tuberculosis* in human macrophages. Sci. Rep. 7, 6484 10.1038/s41598-017-05916-7 28744015 PMC5526930

[52] Wilburn K.M., Fieweger R.A. and Vanderven B.C. (2018) Cholesterol and fatty acids grease the wheels of *Mycobacterium tuberculosis* pathogenesis. Pathogens Dis. 76, fty021 10.1093/femspd/fty021 PMC6251666 29718271

[53] Sassetti C.M. and Rubin E.J. (2003) Genetic requirements for mycobacterial survival during infection. Proc. Natl. Acad. Sci. U.S.A. 100, 12989–12994 10.1073/pnas.2134250100 14569030 PMC240732

[54] Shimono N., Morici L., Casali N., Cantrell S., Sidders B., Ehrt S. et al. (2003) Hypervirulent mutant of *Mycobacterium tuberculosis* resulting from disruption of the mce1 operon. Proc. Natl. Acad. Sci. U.S.A. 100, 15918–15923 10.1073/pnas.2433882100 14663145 PMC307668

[55] Casali N. and Riley L.W. (2007) A phylogenomic analysis of the Actinomycetales mce operons. BMC Genomics 8, 60 10.1186/1471-2164-8-60 17324287 PMC1810536

[56] de La Paz M., Klepp L., Nuñez-García J., Blanco F.C., Soria M., del Carmen García-Polayo M. et al. (2009) Mce3R, a TetR-type transcriptional repressor, controls the expression of a regulon involved in lipid metabolism in *Mycobacterium tuberculosis*. Microbiology 155, 2245–2255 10.1099/mic.0.027086-0 19389781

[57] Crowe A.M., Casabon I., Brown K.L., Liu J., Lian J., Rogalski J.C. et al. (2017) Catabolism of the last two steroid rings in *Mycobacterium tuberculosis* and other bacteria. mBio 8, e00321–17, 10.1128/mBio.00321-17 28377529 PMC5380842

[58] Savvi S., Warner D.F., Kana B.D., McKinney J.D., Mizrahi V. and Dawes S.S. (2008) Functional characterization of a vitamin B12-dependent methylmalonyl pathway in *Mycobacterium tuberculosis:* implications for propionate metabolism during growth on fatty acids. J. Bacteriol. 190, 3886–3895 10.1128/JB.01767-07 18375549 PMC2395058

[59] Griffin J.E., Pandey A.K., Gilmore S.A., Mizrahi V., McKinney J.D., Bertozzi C.R. et al. (2012) Cholesterol catabolism by *Mycobacterium tuberculosis* requires transcriptional and metabolic adaptations. Chem. Biol. 19, 218–227 10.1016/j.chembiol.2011.12.016 22365605 PMC3292763

[60] Yang X., Nesbitt N.M., Dubnau E., Smith I. and Sampson N.S. (2009) Cholesterol metabolism increases the metabolic pool of propionate in *Mycobacterium tuberculosis*. Biochemistry 48, 3819–3821 10.1021/bi9005418 19364125 PMC2771735

[61] Layre E., Cala-De Paepe D., Larrouy-Maumus G., Vaubourgeix J., Mundayoor S., Lindner B. et al. (2011) Deciphering sulfoglycolipids of *Mycobacterium tuberculosis*. J. Lipid Res. 52, 1098–1110 10.1194/jlr.M013482 21482713 PMC3090231

[62] Rhoades E.R., Streeter C., Turk J. and Hsu F.-F. (2011) Characterization of sulfolipids of *Mycobacterium tuberculosis* H37Rv by multiple-stage linear ion-trap high-resolution mass spectrometry with electrospray ionization reveals that the family of sulfolipid II predominates. Biochemistry 50, 9135–9147 10.1021/bi2012178 21919534 PMC3214629

[63] Kumar S., Matange N., Umapathy S. and Visweswariah S.S. (2015) Linking carbon metabolism to carotenoid production in mycobacteria using Raman spectroscopy. FEMS Microbiol. Lett. 362, 1–6 10.1093/femsle/fnu048 25673658

[64] Gouzy A., Poquet Y. and Neyrolles O. (2014) Amino acid capture and utilization within the *Mycobacterium tuberculosis* phagosome. Future Microbiol. 9, 631–637 10.2217/fmb.14.28 24957090

[65] Harper C., Hayward D., Wiid I. and van Helden P. (2008) Regulation of nitrogen metabolism in *Mycobacterium tuberculosis:* a comparison with mechanisms in *Corynebacterium glutamicum* and *Streptomyces coelicolor*. IUBMB Life 60, 643–650 10.1002/iub.100 18493948

[66] Williams K.J., Jenkins V.A., Barton G.R., Bryant W.A., Krishnan N. and Robertson B.D. (2015) Deciphering the metabolic response of *Mycobacterium tuberculosis* to nitrogen stress. Mol. Microbiol. 97, 1142–1157 10.1111/mmi.13091 26077160 PMC4950008

[67] Petridis M., Benjak A. and Cook G.M. (2015) Defining the nitrogen regulated transcriptome of *Mycobacterium smegmatis* using continuous culture. BMC Genomics 16, 821 10.1186/s12864-015-2051-x 26482235 PMC4617892

[68] Gouzy A., Poquet Y. and Neyrolles O. (2014) Nitrogen metabolism in *Mycobacterium tuberculosis* physiology and virulence. Nat. Rev. Microbiol. 12, 729–737 10.1038/nrmicro3349 25244084

[69] Agapova A., Serafini A., Petridis M., Hunt D.M., Garza-Garcia A., Sohaskey C.D. et al. (2019) Flexible nitrogen utilisation by the metabolic generalist pathogen *Mycobacterium tuberculosis*. eLife 8, 1–22 10.7554/eLife.41129 PMC6361586 30702426

[70] Borah K., Beyß M., Theorell A., Wu H., Basu P., Mendum T.A. et al. (2019) Intracellular *Mycobacterium tuberculosis* exploits multiple host nitrogen sources during growth in human macrophages. Cell Rep. 29, 3580–3591 10.1016/j.celrep.2019.11.037 31825837 PMC6915324

[71] Amon J., Titgemeyer F. and Burkovski A. (2009) A genomic view on nitrogen metabolism and nitrogen control in mycobacteria. J. Mol. Microbiol. Biotechnol. 17, 20–29 10.1159/000159195 18824837

[72] Sohaskey C.D. and Wayne L.G. (2003) Role of narK2X and narGHJI in hypoxic upregulation of nitrate reduction by *Mycobacterium tuberculosis*. J. Bacteriol. 185, 7247–7256 10.1128/JB.185.24.7247-7256.2003 14645286 PMC296237

[73] Gouzy A., Larrouy-maumus G., Wu T., Peixoto A., Levillain F., Lugo-villarino G. et al. (2013) *Mycobacterium tuberculosis* nitrogen assimilation and host colonization require aspartate. Nat. Chem. Biol. 9, 674–676 10.1038/nchembio.1355 24077180 PMC3856356

[74] Jansen R.S., Mandyoli L., Hughes R., Wakabayashi S., Pinkham J.T., Selbach B. et al. (2020) Aspartate aminotransferase Rv3722c governs aspartate-dependent nitrogen metabolism in *Mycobacterium tuberculosis*. Nat. Commun. 11, 1960 10.1038/s41467-020-15876-8 32327655 PMC7181641

[75] Gouzy A., Larrouy-Maumus G., Bottai D., Levillain F., Dumas A. et al. (2014) *Mycobacterium tuberculosis* exploits asparagine to assimilate nitrogen and resist acid stress during infection. PLoS Pathog. 10, e1003928 10.1371/journal.ppat.1003928 24586151 PMC3930563

[76] Viljoen A.J., Kirsten C.J., Baker B., van Helden P.D. and Wiid I.J.F. (2013) The role of glutamine oxoglutarate aminotransferase and glutamate dehydrogenase in nitrogen metabolism in *Mycobacterium bovis* BCG. PLoS ONE 8, e84452 10.1371/journal.pone.0084452 24367660 PMC3868603

[77] Awasthy D., Gaonkar S., Shandil R.K., Yadav R., Bharath S., Marcel N. et al. (2009) Inactivation of the *ilvB1* gene in *Mycobacterium tuberculosis* leads to branched-chain amino acid auxotrophy and attenuation of virulence in mice. Microbiology 155, 2978–2987 10.1099/mic.0.029884-0 19542000

[78] Hondalus M.K., Bardarov S., Russell R., Chan J., Jacobs W.R. and Bloom B.R. (2000) Attenuation of and protection induced by a leucine auxotroph of *Mycobacterium tuberculosis*. Infect. Immun. 68, 2888–2898 10.1128/IAI.68.5.2888-2898.2000 10768986 PMC97501

[79] Andries K., Verhasselt P., Guillemont J., Göhlmann H.W., Neefs J.M., Winkler H. et al. (2005) A diarylquinoline drug active on the ATP synthase of Mycobacterium tuberculosis. Science 307, 223–227 10.1126/science.1106753 15591164

